# Receptor interacting protein 3 kinase, not 1 kinase, through MLKL-mediated necroptosis is involved in UVA-induced corneal endothelium cell death

**DOI:** 10.1038/s41420-021-00757-w

**Published:** 2021-11-23

**Authors:** Zhen Yu, Nikolaos E. Efstathiou, Victor S. M. C. Correa, Xiaohong Chen, Kenji Ishihara, Yasuhiro Iesato, Toshio Narimatsu, Dimitrios Ntentakis, Yanyun Chen, Demetrios G. Vavvas

**Affiliations:** 1 Retina Service, Angiogenesis Laboratory, Department of Ophthalmology, Massachusetts Eye and Ear, Harvard Medical School, Boston, MA 02114 USA; 2 Shenzhen Eye Hospital, Shenzhen Key Ophthalmic Laboratory, Jinan University, 518040 Shenzhen, China; 3 Department of Ophthalmology, Retina Service, Massachusetts Eye and Ear, Harvard Medical School, Boston, MA 02114 USA

**Keywords:** Eye diseases, Cell death and immune response

## Abstract

Ultraviolet (UV) is one of the most energetic radiations in the solar spectrum that can result in various tissue injury disorders. Previous studies demonstrated that UVA, which represents 95% of incident photovoltaic radiation, induces corneal endothelial cells (CECs) death. Programmed cell death (PCD) has been implicated in numerous ophthalmologic diseases. Here, we investigated receptor-interacting protein 3 kinase (RIPK3), a key signaling molecule of PCD, in UVA-induced injury using a short-term corneal endothelium (CE) culture model. UVA irradiation activated RIPK3 and mediated necroptosis both in mouse CE and primary human CECs (pHCECs). UVA irradiation was associated with upregulation of key necroptotic molecules (DAI, TRIF, and MLKL) that lie downstream of RIPK3. Moreover, RIPK3 inhibition or silencing in primary corneal endothelial cells suppresses UVA-induced cell death, along with downregulation of MLKL in pHCECs. In addition, genetic inhibition or knockout of RIPK3 in mice (RIPK3^K51A^ and RIPK3^−/−^ mice) similarly attenuates cell death and the levels of necroptosis in ex vivo UVA irradiation experiments. In conclusion, these results identify RIPK3, not RIPK1, as a critical regulator of UVA-induced cell death in CE and indicate its potential as a future protective target.

## Introduction

Ultraviolet light (UV) is one of the most energetic radiations of the solar spectrum, divided into three regions according to wavelength: UVA (320–400 nm), UVB (290–320 nm), and UVC (190–290 nm). Typically, UVA light represents 95% of incident photovoltaic radiation and is absorbed through the entire corneal layer (epithelium, stroma, and endothelium) [1, 2]. As high-energy photon radiation implies a robust biological injury potential in absorbing tissues, exposure to UVA rays is a verified threat in many ocular diseases particularly in corneal endothelium (CE) [2, 3]. The CE is blocked in the postmitotic state and has limited ability to proliferate in vivo. Under regular conditions, even though there is a common loss rate of 0.3–0.6% corneal endothelium cells (CECs) per year, adjacent cells may spread and/or migrate to cover the wound area. The loss of cells beyond the functional reserve threshold causes corneal edema, which requires corneal transplantation to restore vision [4–8]. Therefore, it is important to discover the injury mechanism of UVA to CE and find new protection or treatment methods through it.

Programmed cell death (PCD) includes CASPASE mediated cell death process known as apoptosis and RIPK regulated cell death known as programmed necrosis or necroptosis [9–11]. Although both of these process have been implicated in multiple posterior ophthalmologic diseases such as neovascular age-related macular degeneration (AMD), and retinitis pigmentosa (RP) [12–14], only apoptosis have been implicated in anterior ophthalmic diseases such as Fuchs endothelial corneal dystrophy (FECD) [15, 16]. Once apoptosis activity is decreased then death receptors of necroptosis can be activated [17], followed by activation of receptor-interacting protein kinase 1 (RIPK1 or RIPK1), receptor-interacting protein kinase 3 (RIPK3 or RIPK3), and the mixed lineage kinase domain-like (MLKL) to construct necrosomes that ultimately mediate necroptosis [18, 19].

As a crucial signaling molecule in the pathway of necroptosis, RIPK3 plays an important role in development, tissue injury response, and antiviral immunity [20]. The interaction between RIPK1 and RIPK3, the key elements of the necrosome, gives rise to RIPK3–RIPK3 homo-interaction and RIPK3 auto-phosphorylation. Mixed lineage kinase domain-like (MLKL) is recruited and phosphorylated by RIPK3 [21]. Phosphorylated MLKL then translocates to the cell membrane to execute necroptosis [21–23]. Although both RIPK3 and RIPK1 are required to induce necroptosis, RIPK3 alone can promote necroptosis when overexpressed in cells [24]. When RIPK1 is absent, TIR-domain-containing adapter-inducing interferon-β (TRIF) and DNA-dependent activator of interferon regulatory factors (DAI) are capable to trigger necroptosis through RIP homotypic interaction motif (RHIM)-dependent recruitment of RIPK3 [25, 26]. In addition, the kinase activity of RIPK3 is essential for necroptosis, but it can also determine whether cells may die due to apoptosis [27].

To date, there are no studies investigating the role of RIPK regulated necrosis after UVA injury. In this present study, with the help of genetically engineered mice and inhibitors of RIPK1, RIPK3, and CASPASEs, we explored a critical necroptotic function of RIPK3 in UVA-induced CE injury in vitro and ex vivo.

## Results

### RIPK3 expression increases after high-dose UVA

**Fig. 1:**
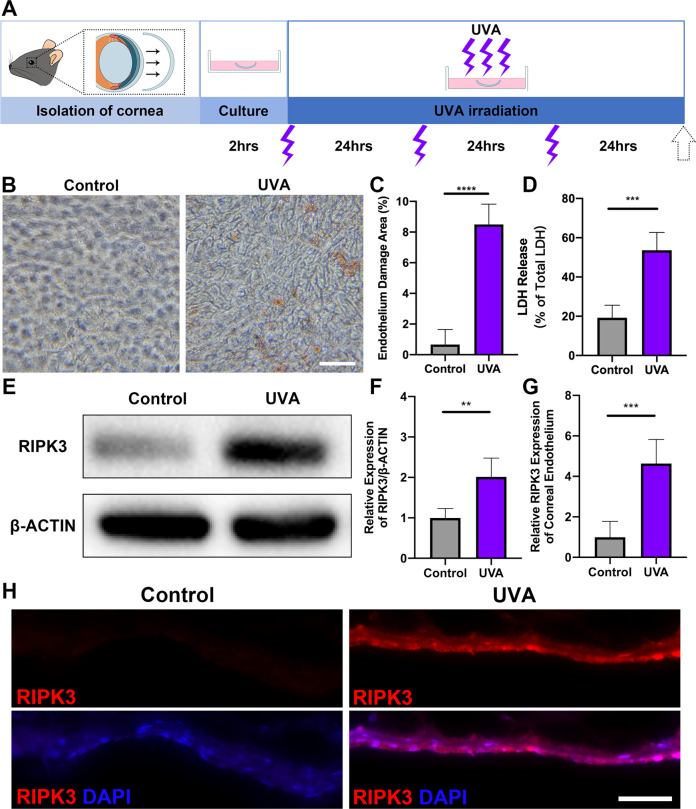
High-dose UVA upregulates the RIPK3 expression in mouse CE. **A** Flow chart of cornea culture ex vivo and UVA irradiation model. The intensity and time of each UVA irradiation were 20 J/cm^2^ (25 min 40 seconds every 24 h) for 72 h. **B** Representative images of Alizarin red S staining in mouse CE from the control and UVA groups (Scale Bar: 20 μm). **C** Endothelium damage area (%) was analyzed by measuring the Alizarin red S-positive area (*n* = 5 per group). **D** LDH release assays of mouse cornea after UVA irradiation (*n* = 5 per group). **E, F** Western blot analysis of RIPK3 expression in mouse CE after UVA irradiation (*n* = 4 per group). **G, H** Representative immunofluorescence images and analysis of RIPK3 (Red) and DAPI (Blue) localization in mouse CE with control and UVA irradiation. (Scale Bar: 50 μm). The fluorescence intensity of RIPK3 under UVA irradiation was compared based on the control group (*n* = 5 per group). Statistical significance was analyzed with the unpaired Student’s *t*-test. **P < 0.01, ***P < 0.001, ****P < 0.0001*.* All values are expressed as mean ± SD.

To evaluate how UVA affects cell death, we set up a UVA injury ex vivo model through the short-term culture of mice CE. After intermittent UVA exposure (25 min 40 seconds every 24 h) for 72 h (Fig. 1A), CE showed disruption of the monolayer as detected by increased Alizarin Red staining of enlarged and irregular cells (8.50 ± 1.32% vs 0.66 ± 0.98%, *P* < 0.0001), as well as increased LDH release (Fig. 1B–D). This was associated with significantly increased expression of RIPK3 detected by western blotting and immunofluorescence (Fig. 1E–H).

### RIPK3, but not RIPK1 is involved in UVA-induced necroptosis

**Fig. 2:**
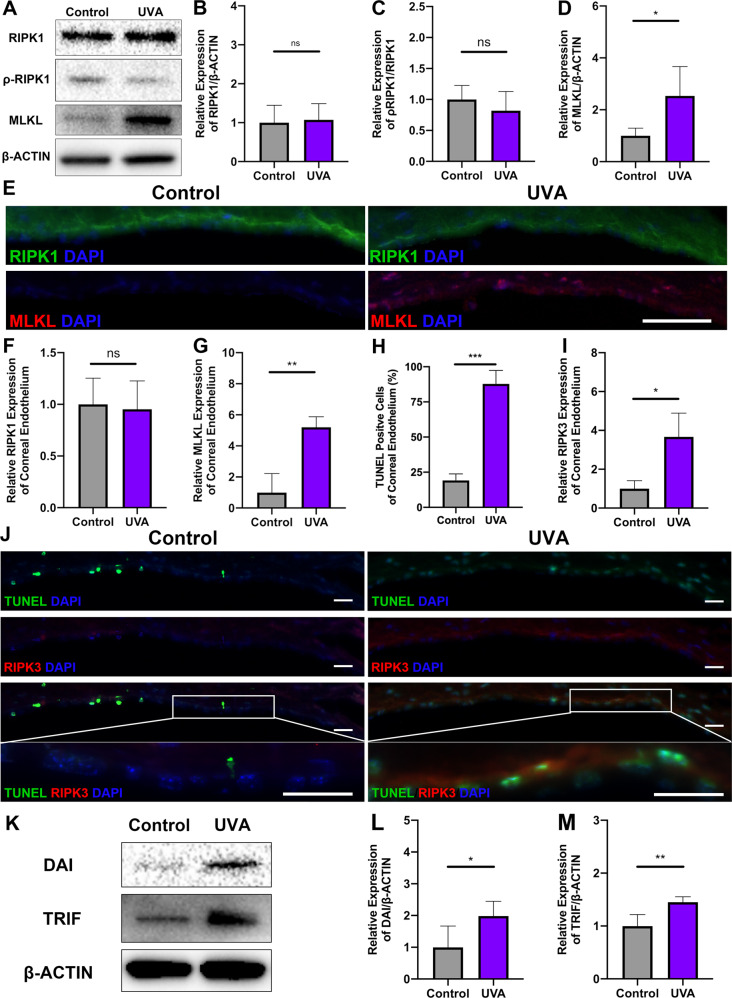
High-dose UVA induced necroptosis, which is not related to RIPK1 in mouse CE. **A**–**D** Western blot analysis of RIPK1, ρ-RIPK1, and MLKL in mouse CE. *n* = 4 per group. **E** Representative immunofluorescence images of RIPK1 (Green), MLKL (Red), and DAPI (Blue) localization in mouse CE with control and UVA irradiation (Scale Bar: 50 μm). **F, G** The fluorescence intensity of RIPK1 and MLKL under UVA irradiation was compared based on the control group (*n* = 3 per group). **H**–**J** TUNEL (Green) and RIPK3 (Red) co-staining after UVA irradiation (Scale Bar: 10 μm). Necroptotic was analyzed by measuring the TUNEL-positive cells (%) and the fluorescence intensity of RIPK3 in mouse CE (*n* = 6 per group). **K**–**M** Western blot analysis of DAI and TRIF in mouse CE (*n* = 4 per group). Statistical significance was analyzed with the unpaired Student’s *t*-test*.* **P < 0.01, ***P < 0.001*,* ****P < 0.0001*.* All values are expressed as mean ± SD.

To further clarify the mechanism of RIPK3 in the CE after UVA irradiation, we examined whether RIPK1, the upstream activator of RIPK3 is also involved in UVA-mediated injury. In contrast to RIPK3, we could not detect any change in either total RIPK1 or phosphorylated RIPK1 (ρ-RIPK1) (Fig. 2A, B). However, other upstream partners of RIPK3 such as DAI and TRIF were significantly upregulated (1.98 ± 0.47-fold, *P* < 0.05 and 1.45 ± 0.10-fold, *P* < 0.01, respectively) (Fig. 2L–M) as well as MLKL, the key downstream protein of RIPK3, (2.54 ± 1.13-fold, *P* < 0.05) (Fig. 2A, D). Immunofluorescence studies confirmed the death of corneal endothelium cells (% of TUNEL-positive cells, 68.64 ± 6.17%, *P* < 0.001) (Fig. 2H, J) and the concomitant upregulation of RIPK3 (3.67 ± 1.22-fold, *P* < 0.05) (Fig. 2I, J) and MLKL (5.19 ± 0.68-fold, *P* < 0.01) (Fig. 2E, G). This suggested that necroptosis pathway is of importance after UVA irradiation of CE.

### RIPK3 inhibition suppresses cell death in the UVA model of pHCECs

**Fig. 3:**
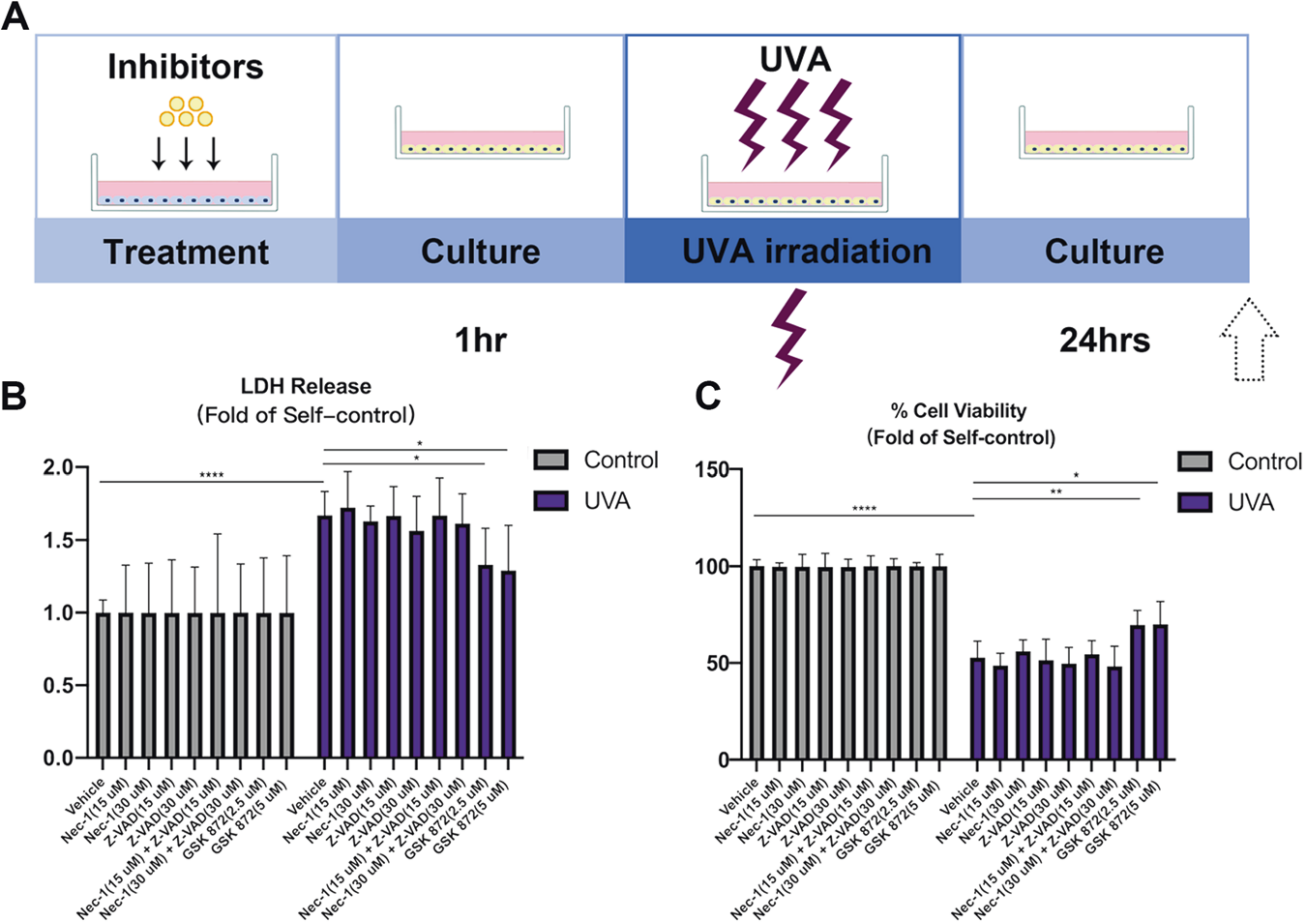
GSK 872, the inhibitor of RIPK3 reduces cell death in pHCECs. **A** Flow chart of pHCECs treatment and UVA irradiation model. **B, C** LDH and Cell viability of pHCECs treated with vehicle DMSO (30 μM), RIPK1 inhibitor Nec-1 (15 and 30 μM), CASPASE inhibitor Z-VAD (15 and 30 μM), combination of Nec-1 and Z-VAD (15 and 30 μM), RIPK3 inhibitor GSK 872 (2.5 and 5 μM) after non-UVA and UVA irradiation (*n* = 6 per group). Statistical significance was analyzed with the unpaired Student’s *t*-test*.* **P < 0.01*,* ***P < 0.001*,* ****P < 0.0001*.* All values are expressed as mean ± SD.

To further explore the role of RIPK3 in UVA CE mediated injury we used various small molecule inhibitors (RIPK1–Nec-1, Pan-CASPASE–Z-VAD-FMK, RIPK3–GSK 872) to assess cell viability after UVA exposure (Fig. 3A). LDH release and MTT assays showed that neither Nec-1(RIPK1 inhibitor) nor Z-VAD (pan-CASPASE inhibitor), nor Nec-1/Z-VAD combination, but only RIPK3 inhibitor GSK 872 was able to decrease UVA-induced cell lethality (Fig. 3B, C).

**Fig. 4:**
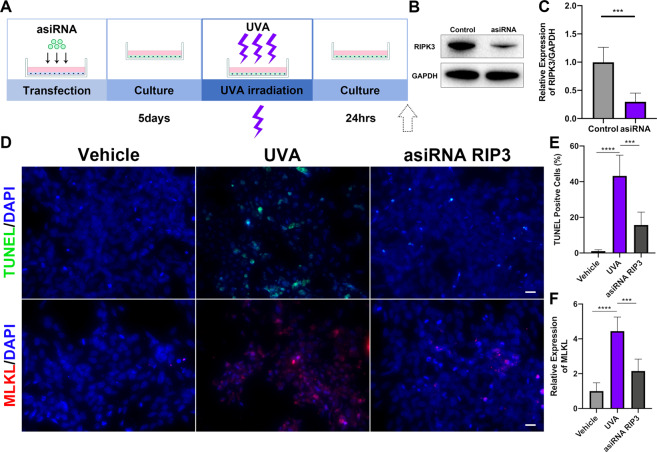
Silencing of RIPK3 suppresses cell death via necroptosis pathway in pHCECs. **A** Flow chart of pHCECs transfection, culture, and UVA irradiation model. **B, C** Western blot analysis of RIPK3 in control and asiRNA RIPK3 transfected pHCECs. **D** Representative images of TUNEL (Green), MLKL (Red), and DAPI (Blue) staining in vehicle, UVA, asiRNA RIPK3 transfected pHCECs (Scale Bar: 20 μm). **E, F** The TUNEL-positive cells (%) and MLKL fluorescence intensity in normal pHCECs and UVA irradiation pHCECs were quantified (a total of 60–200 cells were selected for statistics in each group, *n* = 6 per group). Statistical significance was analyzed with the unpaired Student’s *t*-test*.* **P < 0.01, ***P < 0.001*,* ****P < 0.0001*.* All values are expressed as mean ± SD.

To further examine the role of RIPK3 as a mediator of UVA-induced cell death, we used asiRNA to silence RIPK3 expression (Fig. 4A). After transfection, RIPK3 was downregulated (69.91 ± 10.03%, *P* < 0.001) (Fig. 4B, C) and death decreased (27.58 ± 5.57%, *P* < 0.001) (Fig. 4D, E). This decrease in cell death was also associated with a downregulation of the RIPK3 effector MLKL (2.29 ± 0.43-fold, *P* < 0.001) (Fig. 4D, F).

### Corneal endothelium from RIPK3^K51A^ and RIPK3^−/−^ but not from RIPK1^K45A^ mice have strong resistance to UVA irradiation in an ex vivo model

**Fig. 5:**
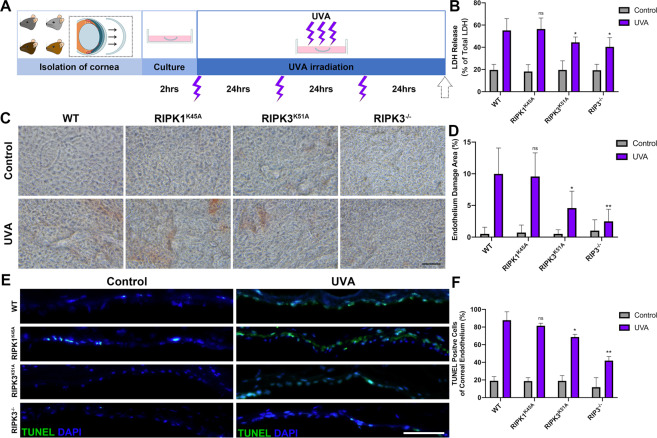
The inhibition of RIPK3 protein expression attenuates cell death after UVA irradiation in transgenic mice CE. **A** Flow chart of Wild-type, RIPK1^K45A^, RIPK3^K51A^, RIPK3^−/−^ cornea culture ex vivo and UVA irradiation model. **B** LDH release assays of transgenic mice CE after UVA irradiation (*n* = 6 per group). **C** Alizarin red S staining in transgenic mice CE with control and UVA irradiation (Scale Bar: 20 μm). **D** Endothelium damage area (%) was analyzed by measuring the Alizarin red S-positive area (*n* = 5 per group). **E** Representative immunofluorescence images of TUNEL (Green) and DAPI (Blue) in transgenic mice CE (Scale Bar: 10 μm). **F** TUNEL-positive cells (%) were quantified and compared among transgenic mice (*n* = 3 per group). Statistical significance was analyzed with the unpaired Student’s *t*-test. **P < 0.01*,* ***P < 0.001*,* ****P < 0.0001*.* All values are expressed as mean ± SD.

Finally, to further evaluate the role of RIPK in UVA-induced injury, corneas from three transgenic lines (RIPK1 kinase dead, RIPK3 kinase dead and RIP3 knockout) were exposed to UVA (Fig. 5A) and cell death and injury were assessed by three methods (LDH release, Alizarin Red and TUNEL staining) (Fig. 5B–F). Corneas from RIPK1 kinase dead (RIPK1^K45A^) mice were not protected from UVA-induced injury. However, corneas from RIPK3 kinase dead (RIPK3^K51A^) and RIPK3^−/−^ mice had less cell death after UVA irradiation further supporting the role of RIPK3 but not RIPK1 in UVA-induced injury of corneal endothelium.

## Disscussion

UV radiation is one of the most prevalent environmental factors influencing human health and disease including eye diseases. UVA penetrates all corneal layers including endothelium and has recently been implicated in the pathogenesis of Fuchs corneal dystrophy endothelium [3, 28, 29]. However, the mechanism of UVA damage to CE has not been fully elucidated. Here, we applied the UVA irradiation in the short-term culture of mouse whole cornea and identified a significant role for RIPK3 but not RIPK1 mediated programmed cell death. The connection between UV irradiation and upregulation of RIPK has not been reported before for any cell type and establishes a novel connection between UV irradiation and cell injury at least in the corneal endothelium. This was also confirmed in the primary human cultured corneal endothelial cells (pHCECs) UVA model (Fig. S1).

**Fig. 6:**
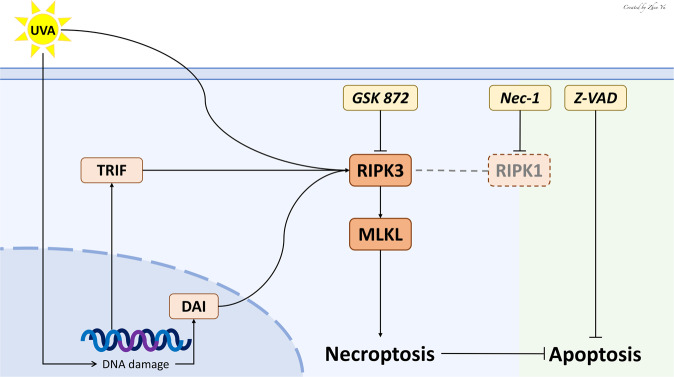
Schematic summary for the present study. UVA-induced DNA damage leads to RIPK3 activation through the TRIF and DAI signal responses. RIPK3 but not RIPK1 in turn induces necroptosis by upregulation of MLKL expression and suppresses apoptosis by downregulation of GSK 872 (RIPK3 inhibitor) also can positively regulate UVA-induced necroptosis, while Nec-1 (RIPK1 inhibitor) and Z-VAD (apoptosis inhibitor) cannot.

RIPK1/3 are universally expressed and widely known for their role in promoting cell death, especially necroptosis [30, 31]. Necroptosis is regulatory necrosis that heavily relies on RIPK1/3-mediated complexes containing MLKL. In particular is thought that the activation of RIPK3 leads to MLKL oligomerization and phosphorylation, and eventually the execution of necroptosis [20, 32, 33]. Classically RIPK mediated programmed cell death involves the activation of cell death receptors and recruitment of RIPK1 in the necrosome, especially in the presence of CASPASE inhibition [34, 35]. The activated RIPK1 phosphorylates and activates RIPK3 [36]. However, in the model of UVA-induced cell death, we did not observe RIPK1 upregulation or activation. Furthermore, small molecule inhibitors of RIPK1 (Nec-1) did not protect the CE [37]. Whereas GSK 872 protected the CE, which is a cell-permeable quinolinyl-benzothiazolamine compound that is reported to act as a RIPK3-selective kinase inhibitor with >1000-times selectivity over a vast majority of more than 300 other kinases, including RIPK1 [38–40]. To validate this further we used endothelium for mice with kinase dead RIPK1 [41], RIPK3 [38] and null for RIPK3 [42]. Consistent with the small molecule experiments, endothelium from RIPK1 kinase dead animals did not exhibit altered UVA-induced cell death, whereas the absence of RIPK3 in CE or presence of kinase dead RIPK3, led to protection from UVA damage. Thus, we can conclude that RIPK3 inhibition or deficiency represses UVA-mediated cell necroptosis, while RIPK1 is not involved in UVA-induced necroptosis. This is in line with other work that has shown that RIPK3 can be activated by non RIPK1-dependent mechanisms [43, 44]. It has been shown that intracellular stressors can lead to activation of RIPK3 through mechanisms involving TRIF and/or DAI[45]. The adaptor protein TRIF contains one RHIM, and the innate immune sensor DAI contains three putative RHIMs, which is essential for binding with RIPK3 to trigger the necroptotic process [26, 46, 47]. In the present study, the expression of DAI and TRIF were both upregulated (Fig. 2K-M) supporting that RIPK3 may be activated after UVA exposure by the upstream signals of DAI and TRIF. Based on these data, we propose a summary scheme of RIPK mediated UVA-induced cell death in Fig. 6.

Suppression of RIPK3 has potential for UVA protection. Small interfering RNA (siRNA) is short double-stranded RNA and mediates RNA interference (RNAi) that leads to eukaryotic gene suppression [48, 49]. However, the main barrier that needs to be overcome in the development of RNAi therapy using siRNA molecules to effectively deliver to diseased cells or tissues continues to be that the phosphate backbone structure prevents siRNA from entering the cell through the plasma membrane. Asymmetric small interfering RNA (asiRNA) can enter cells spontaneously and trigger RNAi-mediated gene silencing without complex conveying systems [50]. pHCECs directly transfected with RIPK3 asiRNA showed an efficient reduction of RIPK3 expression and protection from cell death.

In summary, our results reveal for the first time the role of RIPK3 mediated necroptosis in UVA-induced cell death of corneal endothelium and suggest that suppression of RIPK3 may be a potential target for UVA-induced injury. In contrast, to RIPK3, upstream activator RIPK1 did not appear to play a role in CE cell necroptosis after UVA irradiation. It would be of interest to explore more mechanisms of UVA-induced DAI and TRIF activation and explore more effective and specific protective mechanisms in UVA-induced CE injury in vivo.

## Materials and methods

### Animals

All animal procedures complied with the Association for Research in Vision and Ophthalmology (ARVO) Statement for the Use of Animals in Ophthalmic and Vision Research under the approval by the Animal Care Committee of Massachusetts Eye and Ear Infirmary. RIPK1^K45A^ and RIPK3^K51A^ mice were kindly provided by GlaxoSmithKline. RIPK3^−/−^ mice and wild-type C57BL/6 J mice were purchased from the Jackson Laboratory. All the mice were fed chow and water on standard laboratory diet with a 12 h light/12 h dark cycle in an air-controlled room.

### Whole mouse corneal isolation and ex vivo culture

After mice euthanization, eyes were enucleated and immersed in phosphate-buffered saline (PBS) (Gibco, CA, USA) for 15 seconds. A small incision was made at the limbus through a 30 G needle, under the microscope and was followed by a circular-cut along the limbus with 3 mm cutting edge spring scissors. Immediately after removing the iris ring, the corneal cups were dissected and trimmed of non-corneal tissue in a 60 mm sterile petri dish filled with PBS to harvest the pure whole corneas. Subsequently, the separated corneas were cultured in a 24-well plate in the presence of pre-warmed culture medium. Culture medium was prepared by adding 5% nonessential amino acids (Gibco), 40 μL 99% β-Mercaptoethanol (Sigma–Aldrich, MO, USA), 40 μL insulin-transferrin-Selenium (Gibco), 500 μg hydrocortisone (Sigma–Aldrich), penicillin-streptomycin (Gibco), and 10% heat-inactivated FBS (Gibco) to 500 mL Dulbecco’s Modified Eagle Medium (DMEM) (Gibco). Medium was replaced with fresh after 2 h of culture. Corneas were culture in a humidified 5% CO_2_ incubator at 37 °C.

### Mouse cornea UVA irradiation model

The corneas were irradiated with UVA or not 2 h after the medium was replaced. Two UVA tubes (365 nm wavelength, XX-15BLB; Analytik Jena, MA, USA) were used for irradiation at a distance of 7 cm. The irradiation dose was 20 J/cm^2^ each time with a radiation intensity of 12.99 mW/cm^2^ for 25 min and 40 seconds, which was measured by a UVA sensor (Fisher Scientific, MA, USA). The subsequent two irradiations were separated by 24 h, and the irradiation conditions were the same as the first. The corneas were harvested 24 h after the last irradiation (Fig. 1A).

### Alizarin Red S staining

After UVA irradiation, corneas were harvested and washed twice with warm PBS, and soaked in 8 mM Alizarin Red S solution (Sigma–Aldrich) diluted with distilled water for 90 seconds. After washing twice again with PBS, corneas were fixed with 4% paraformaldehyde (PFA) for 10 min, and then washed again in PBS three times (5 min each). Corneas were mounted on glass slides and images were captured with an inverted brightfield microscope (Primeovert, Zeiss, NY, USA).

### Primary human CECs culture (pHCECs) and UVA model

pHCECs (Celprogen, CA, USA) were seeded at a concentration of 2.0 × 10^5^ cells/mL in six-well plates and cultured in the pHCECs medium supplemented with Serum Media (M36081-13S, Celprogen). The UVA model was induced by two 365 nm wavelength UVA tubes (Analytik Jena) at a distance of 7 cm. The total irradiation energy was 5 J/cm^2^ (6 min 25 seconds), 10 J/cm^2^ (12 min 50 seconds), and 20 J/cm^2^ (25 min 40 seconds). The irradiation doses were measured by a UVA sensor (Fisher Scientific). The cells were harvested 24 h after irradiation (Fig. S1A).

### Scratch assay

pHCECs were seeded at a concentration of 2.0 × 10^5^ cells/mL in 12-well plates and cultured for 48 h. After reaching confluence, wounds were created by scratching with a 200 μL sterile pipette tip. Gently rinse the well plate twice with warm PBS to remove the scraped out cells and add the culture medium. The cells were immediately irradiated with different doses and images were captured at 0 h, 12 h, 24 h, 36 h with an inverted brightfield microscope (Zeiss). Image J software (Wayne Rasband, National Institutes of Health, MD, USA) was used to measure the wound area.

### Lactate dehydrogenase (LDH) cytotoxicity assay

LDH release assay was performed following the manufacturer’s protocol (LDH Cytotoxicity Assay Kit; Cell Biolabs, CA, USA). Briefly, RIPK1 inhibitor Necrostatin-1 (NEC-1) (15 or 30 μM) (N9037, Sigma–Aldrich), Pan-CASPASE inhibitor Z-VAD (15 or 30 μM) (FMK001, R&D Systems, MN, USA), the combination of Nec-1 and Z-VAD (both 15 or 30 μM), RIPK3 inhibitor GSK 872 (2.5 or 5 μM) (5303890001, Sigma–Aldrich), and DMSO vehicles (Sigma–Aldrich) were added in the culture medium 1 h before UVA irradiation. Twenty four hours post UVA irradiation, with 15 μL triton X-100 solution was added for 10 min, in the wells that were set as a positive control. Ninety microliters of cell culture medium and 10 μL LDH substrate were transferred and mixed into a 96-well plate. The mixture was incubated at 37 °C and 5% CO_2_ in the dark until the OD 450 values of positive control wells no longer increased. The 96-well plate was read at 450 nm wavelength using an absorbance microplate reader (Molecular Devices, CA, USA). The levels of LDH release were standardized with the positive control as 100% cell death.

### Cell viability assay

MTT cell proliferation assay kit (MTT Cell Proliferation Assay; Cell Biolabs) was used for testing pHCECs viability following the manufacturer’s protocol. In short, the pHCECs were seeded at a concentration of 2.0 × 10^4^ cells/mL in 96-well plate. Cells were cultured for 48 h at 37 °C and 5% CO2 in a humidified incubator. Nec-1 (15 or 30 μM), Z-VAD (15 or 30 μM), the combination of Nec-1 and Z-VAD (both 15 or 30 μM), RIPK3 inhibitor GSK 872 (2.5 or 5 μM), and DMSO vehicles were added in the culture medium 1 h before UVA irradiation (Fig. 3A). Ten microliters of the CytoSelectTM MTT Cell Proliferation Assay Reagent was added to each well 24 h post UVA irradiation. Then, the plate was incubated at 37 °C and 5% CO_2_ for 2 h until purple precipitate was visible under microscope. After adding 100 μL of Detergent Solution into each well, the plate was incubated for additional 2 hours protected from light. The plate was read at 545 nm wavelength using an absorbance microplate reader (Molecular Devices).

### asiRNA transfection

Equal amounts of equimolar sense and anti-sense strands of RIPK3 asiRNA (OliX Pharmaceuticals, Inc., MA, USA) were mixed in Opti-MEM (Gibco) to create duplex asiRNAs as described before [51]. pHCECs were transfected with RIPK3 asiRNA in the final concentration of 1000 nM for 5 days prior to experiments (Fig. 4A).

### Immunofluorescence and TUNEL staining

The irradiated corneas were rinsed in warm PBS twice and embedded in OCT compound for cryosections. The corneal samples were cryosectioned at 8 μm thickness. For pHCECs, cells were cultured in a sterile 8-well glass slide (Sigma–Aldrich) and irradiated by UVA based on above description (see UVA model). Tissue or cell slides were fixed in 4% PFA for 15 min, blocked with 1% bovine serum albumin (BSA) with 0.4% Triton X-100 in PBS for 1 h at room temperature (RT). The slides were incubated with primary antibodies (Table S1) overnight at 4 °C. Subsequently, the slides were washed with PBS for 5 min three times, incubated with secondary antibodies (Thermo Fisher Scientific, Waltham, MA, USA) for 1 h at RT, washed three times with PBS, and coverslipped using PloLong Gold Antifade Reagent (Thermo Fisher Scientific). For TUNEL staining, the assay was performed by using the ApopTag Plus In Situ Apoptosis Fluorescein Detection Kit (S7111, Thermo Fisher Scientific). Images were captured using a microscope Axio imager M2 (M2, Zeiss, Oberkochen, Germany).

### Western blot

After UVA irradiation, the CE layer was isolated by removing corneal epithelium and stroma under microscope. The harvested CE layer or cells were, respectively, homogenized in prechilled tissue protein extraction reagent (T-PER, Thermo Fisher Scientific) or mammalian protein extraction reagent (M-PER, Thermo Fisher Scientific) both with protease/phosphatase inhibitor cocktail (cOmplete Mini, IN, USA). Protein concentration was measured by Coomassie (Bradford) Protein Assay Kit (Thermo Fisher Scientific). The samples were heated in NuPAGE Sample Buffer (Thermo Fisher Scientific) containing 5% Tris (2-carboxyethyl) phosphine hydrochloride solution (Sigma–Aldrich) at 95 °C for 5 min. Electrophoresis performed with NuPAGE Bis-Tris Gels (Thermo Fisher Scientific) at 120 V. Next, the transferred polyvinylidene difluoride membranes (Sigma–Aldrich) were blocked and incubated with primary antibodies at 4 °C overnight followed by labeling with HRP-conjugated secondary antibodies at room temperature for 1 h. The incubated membranes were developed with HRP substrate reagent (Genetex, CA, USA) and recorded with an ECL imaging system ChemiDoc MP (Bio-Rad Laboratories, CA, USA).

### Statistical analysis

All experiments were performed at least three times and data are presented as the mean of these independent experiments. Statistical analyses were performed by Prism software (GraphPad 8.0, CA, USA). Results were described as mean ± SD. Unpaired Student *t*-test was used to analyze statistical differences between two groups. ANOVA followed with post-hoc Tukey HSD test was used to multiple groups. The significance differences were defined as *P* < 0.05 (*), *P* < 0.01 (**), and *P* < 0.001 (***).

## Supplementary information

Table S1

Figure legend S1

Figure S1

## Data Availability

The datasets used and/or analyzed during the current study are available from the corresponding author on reasonable request.
