# Supplementary material for: Receptor interacting protein 3 kinase, not 1 kinase, through MLKL-mediated necroptosis is involved in UVA-induced corneal endothelium cell death
Source: Cell Death Discov. 2021 Nov 23;7:366. doi: 10.1038/s41420-021-00757-w (PMC8611008; doi:10.1038/s41420-021-00757-w)
Supplement: Supplementary file 1 — Table S1 [file 41420_2021_757_MOESM1_ESM.docx]

**Table S1**. Antibodies for immunofluorescence and western blot used in this study.

| **Antigen** | **Company** | **Catalog #** | **Dilution (IF)** | **Dilution (WB)** |
| --- | --- | --- | --- | --- |
| RIP3 | NOVUS | NBP1-77299 |  | 1:1 000 |
| RIP3 | SIGMA | R4277 | 1:200 |  |
| RIP1 | BD Biosciences | 610459 | 1:200 | 1:1 000 |
| ρ-RIP1 | Thermo Fisher | PA5-105640 |  | 1:1 000 |
| MLKL | Biorbyt | orb32399 | 1:200 | 1:800 |
| β-ACTIN | CST | 4970S |  | 1:1 000 |
| DAI | NOVUS | NBP1-76854 |  | 1:1 000 |
| TRIF | NOVUS | NB120-13810 |  | 1:1 000 |
