# Supplementary material for: Receptor interacting protein 3 kinase, not 1 kinase, through MLKL-mediated necroptosis is involved in UVA-induced corneal endothelium cell death
Source: Cell Death Discov. 2021 Nov 23;7:366. doi: 10.1038/s41420-021-00757-w (PMC8611008; doi:10.1038/s41420-021-00757-w)
Supplement: Supplementary file 2 — Figure legend S1 [file 41420_2021_757_MOESM2_ESM.docx]

**Supplemental Materials**

**Fig. S1 RIPK3 in primary human CECs (pHCECs) is activated by high-dose UVA irradiation. A** Flow chart of pHCECs *in vivo* culture and UVA irradiation model. The intensity and time of UVA irradiation were 5 J/cm^2^ (6 minutes 25 seconds), 10 J/cm^2^ (12 minutes 50 seconds) and 20 J/cm^2^ (25 minutes 40 seconds). **B** Migration during 36 hours after UVA irradiation with the intensity of 0, 5, 10, 20 J/cm^2^ (Scale Bar: 100 μm). **C** The proliferation rate of pHCECs slowed down with the increase of UVA irradiation intensity by analyzing the wound area (n=6 per group). **D & E** Western blot analysis of RIPK3 expression in pHCECs with UVA irradiation (n=5 per group). **F** Representative immunofluorescence images of relative RIPK3 (Red) and DAPI (Blue) in pHCECs with UVA irradiation (Scale Bar: 20 μm). **G** The average fluorescence intensity of RIPK3 protein in pHCECs with UVA irradiation (a total of 100-200 cells were selected for statistics in each group, n=3 per group *Statistical significance was analyzed with the unpaired Student's t-test. **P < 0.01, ***P < 0.001, ****P < 0.0001. All values are expressed as mean ± SD.*
