# Supplementary figures and images for: Receptor interacting protein 3 kinase, not 1 kinase, through MLKL-mediated necroptosis is involved in UVA-induced corneal endothelium cell death
Source: Cell Death Discov. 2021 Nov 23;7:366. doi: 10.1038/s41420-021-00757-w (PMC8611008; doi:10.1038/s41420-021-00757-w)

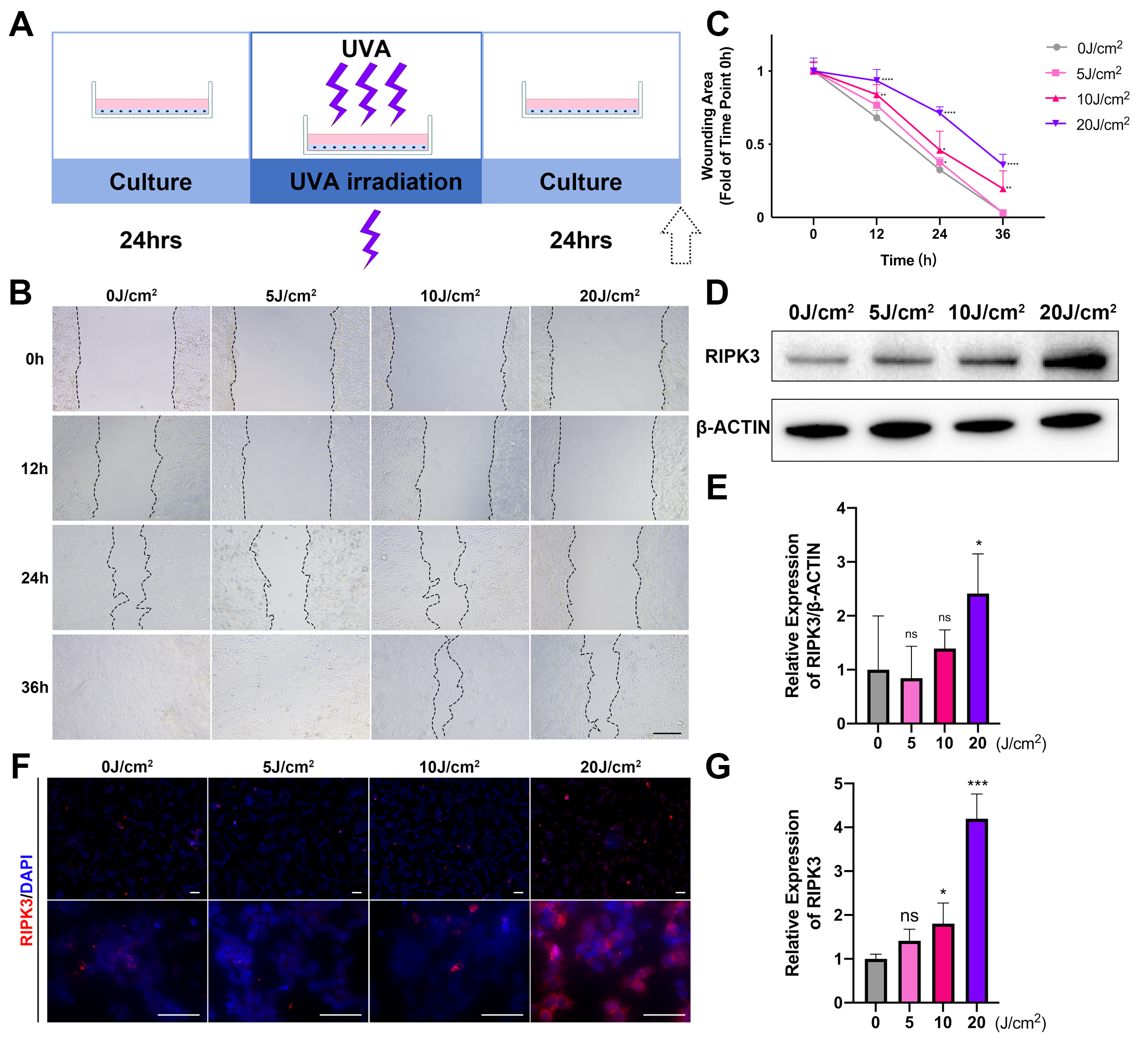

Supplement: Supplementary file 3 — Figure S1 [file 41420_2021_757_MOESM3_ESM.tif]
